# Molecular Detection and Phylogenetic Analysis of Tick-Borne Pathogens in Ticks Collected from Horses in the Republic of Korea

**DOI:** 10.3390/pathogens10091069

**Published:** 2021-08-24

**Authors:** Hyun-Ji Seo, A-Tai Truong, Keun-Ho Kim, Ji-Yeon Lim, Subin Min, Heung-Chul Kim, Mi-Sun Yoo, Soon-Seek Yoon, Terry A. Klein, Yun Sang Cho

**Affiliations:** 1 Parasitic and Honeybee Disease Laboratory, Bacterial Disease Division, Animal and Plant Quarantine Agency, Gimcheon 39660, Korea; hyunj3589@korea.kr (H.-J.S.); or taita@tnus.edu.vn (A.-T.T.); northbear5@korea.kr (K.-H.K.); jiyeom75@hanmail.net (J.-Y.L.); alstnqls5917@naver.com (S.M.); msyoo99@korea.kr (M.-S.Y.); yoonss24@korea.kr (S.-S.Y.); 2 Faculty of Biotechnology, Thai Nguyen University of Sciences, Thai Nguyen 2500000, Vietnam; 3 Force Health Protection and Prevention Medicine, Medical Department Activity-Korea, 65th Medical Brigrade, Unit 15281, APO AP 96271-5281, USA; hungchol.kim2.ln@mail.mil (H.-C.K.); terry.a.klein2.civ@mail.mil (T.A.K.)

**Keywords:** horse ticks, tick-borne pathogens, *Anaplasma phagocytophilum*, *Borrelia* spp., *Haemaphysalis longicornis*, *Ixodes nipponensis*

## Abstract

The horse industry has grown rapidly as a leisure industry in the Republic of Korea (ROK) in parallel with an increased demand for equestrian activities. As a result, there has been an increase in horse breeding and equestrian population and potential exposure to ticks and their associated pathogens. To provide a better understanding of the potential disease risks of veterinary and medical importance, a study was conducted to determine the geographical distribution and diversity of ticks collected from horses and vegetation associated with horse racetracks/ranches throughout the ROK. This included a survey of five associated common pathogens, *Anaplasma phagocytophilum*, *Ehrlichia chaffeensis*, *Borrelia* spp., *Babesia caballi*, and *Theileria equi*. A total 9220 ticks were collected from horses and associated pastures. Ticks were identified to species, stage of development, and sex. Two species of ticks, *Haemaphysalis longicornis* (99.9%) and *Ixodes nipponensis* (0.1%) were identified. Two of the target pathogens, *A. phagocytophilum* and *Borrelia* spp., were detected in 5/1409 tick pools (0.35%) and 4/1409 pools (0.28%) of *H. longicornis*, respectively, both of which are zoonotic pathogens of medical importance. The results of 16S rRNA phylogenetic analysis of *A. phagocytophilum* showed a close relationship to strains distributed in China, USA, Germany, Italy, Turkey, and Poland. *Borrelia* spp. showed a close relationship, based on 16S rRNA gene, to the strains reported from the USA (*B. burgdorferi* and *B. americana*) and Japan (*B. tanukii* and *B. garinii*). These results provide information about the potential risks of veterinary and medical importance and the development of mitigation strategies for disease prevention.

## 1. Introduction

The number of horses has grown rapidly following the enactment of the Horse Industry Promotion Act by the Republic of Korea (ROK) government in 2011. The Korean government invested 600 billion won (USD 543,133,883) from 2011–2018 to foster the horse industry [1,2]. By 2019, there were 27,246 horses, 459 horseback riding facilities, and 919,556 riders in the ROK, with increases of 11.4%, 38.7% and 18.1%, respectively, compared to 2013 data [1]. The higher numbers of horses, associated horse facilities, and riders have increased the potential for exposure to ticks and transmission of tick-borne pathogens to both horses and associated equestrian personnel.

A wide range of wild (deer, raccoon dogs, brown bears, red fox, gray wolves) and domestic animals (horses, cattle, dogs, sheep) are hosts of ticks that harbor tick-borne pathogens, e.g., *Babesia*, *Theileria*, *Anaplasma*, and *Borrelia* species [3,4,5,6,7]. The prevalence of horse ticks varies according to geographical regions, such as *Amblyomma cajennense* and *Anocentor nitens* in Brazil [8], *Amblyomma americanum*, *Dermacentor variabilis*, *Amblyomma maculatum* in Oklahoma, US [9], and 13 species belonging to Ixodidae Family in Italy [10]. Ticks and tick-borne diseases (TBD) associated with horses have been reported in different countries (Italy, Spain, Sweden, Guatemala, Taiwan) where selected pathogens have resulted in abortion and decreased animal production [3,11,12,13,14]. Additionally, horse ranch personnel and riders are exposed to biting ticks and associated transmission of tick-borne pathogens [15,16].

In the ROK, *Haemaphysalis longicornis* harbors various pathogens, e.g., *Candidatus* Rickettsia longicornii, *Ehrlichia canis*, and *Theileria luwenshuni* that have been reported in horses at Jeju Island [17]. However, investigations on the distribution of ticks and associated pathogens that are associated with horses and pasture lands in the ROK have not been conducted. The aims of this study were to identify tick species associated with horses and horse ranches at three metropolitan cities and seven provinces in the ROK, and to detect selected tick-borne pathogens: *A. phagocytophilum*, *E. chaffeensis*, *Borrelia* spp., *B. caballi*, and *T. equi*.

## 2. Results

### 2.1. Prevalence of Ticks Infesting Horses in ROK

A total 9220 ticks consisting of 2267 larvae; 5433 nymphs; and 1520 adults (412 male and 1108 female) were collected during 2016 and 2017, in which 1686 ticks (18.3%) were collected from horses (1090 adults, 595 nymphs, 1 larva) and 7534 ticks (81.7%) from vegetation associated with racetracks/ranches (430 adults, 4838 nymphs, 2266 larvae) (Table 1). A total of 1532 ticks (16.6%); 2184 ticks (23.7%), and 5504 ticks (59.7%) were collected at racetracks under the Racing Horse Authority (RHA), private horse farms (PHF), and leisure horse-riding ranches (LHR), respectively (Table 2). The largest number of ticks were collected from Jeju Island (6633; 71.9%), followed by Gyeonggi (614; 6.7%) and Jeollanam (380; 4.1%) provinces (Table 1). Nymphs accounted for the highest number of ticks collected (5433; 58.9%), followed by larvae (2267; 24.6%) and adults (1520; 16.5%) (Table 1).

**Figure 1:**
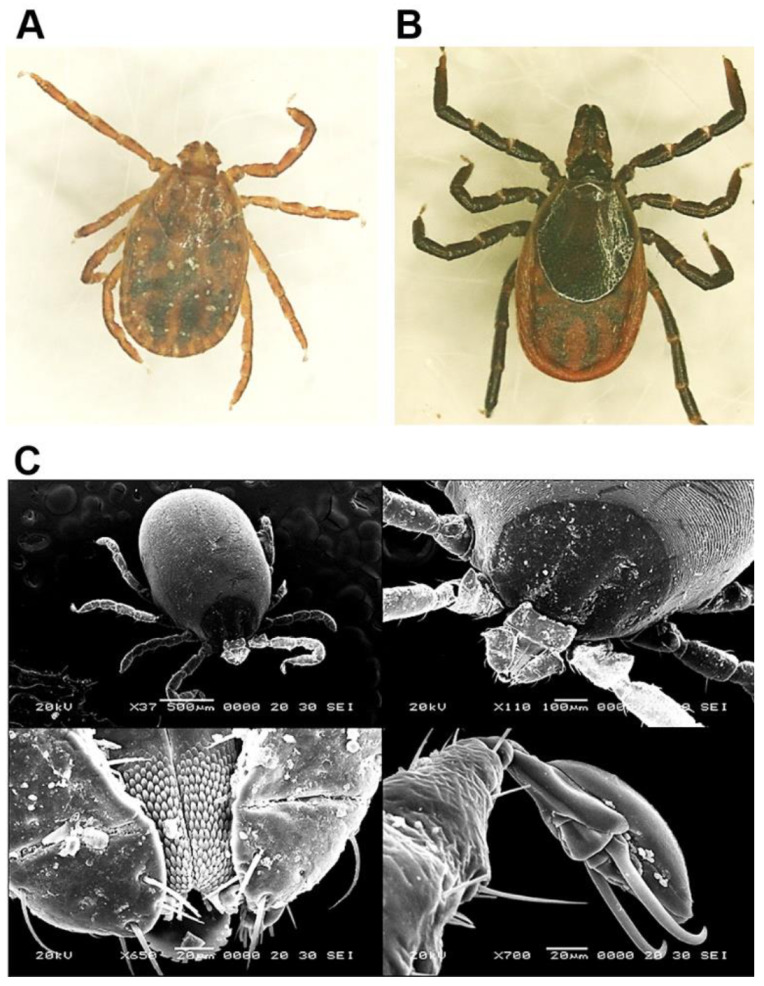
Morphological characterization of *H. longicornis* and *I. nipponensis* using light microscopy and scanning electron microscopy. (**A**) *H. longicornis* and (**B**) *I. nipponensis* females observed under a light microscope at 40 × magnification. (**C**) Characterization of *H. longicornis* was performed using a scanning electron microscope.

Only two species of ticks, *Haemaphysalis longicornis* (9214; 99.9%) and *Ixodes nipponensis* (6; 0.1%) were collected directly from horses and associated vegetation at racetracks and horse ranches (Table 1; Figure 1). *Haemaphysalis longicornis* was collected from all 10 areas, while *I. nipponensis* was only collected at Gyeonggi and Jeollabuk provinces.

### 2.2. Detection of Tick-Borne Pathogens

**Figure 2:**
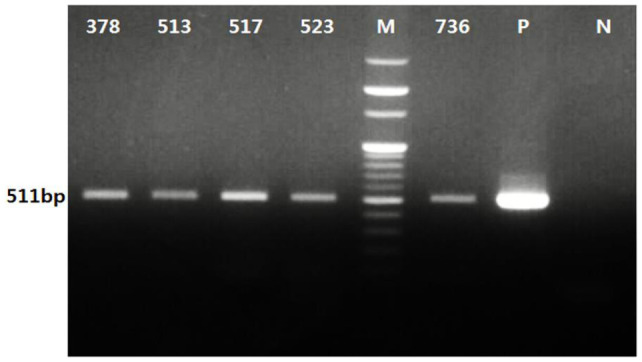
PCR analysis of *A. phagocytophilum* from ticks collected from horses and associated vegetation in the Republic of Korea (ROK). Positive detection of *A. phagocytophilum* in five tick pools (378, 513, 517, 523, and 736 ticks) was confirmed with an expected 511 bp band observed using electrophoresis. “P” and “N” represent a positive control using recombinant *A. phagocytophilum* DNA and a negative control without a DNA template, respectively. “M” represents a 100 bp DNA marker.

**Figure 3:**
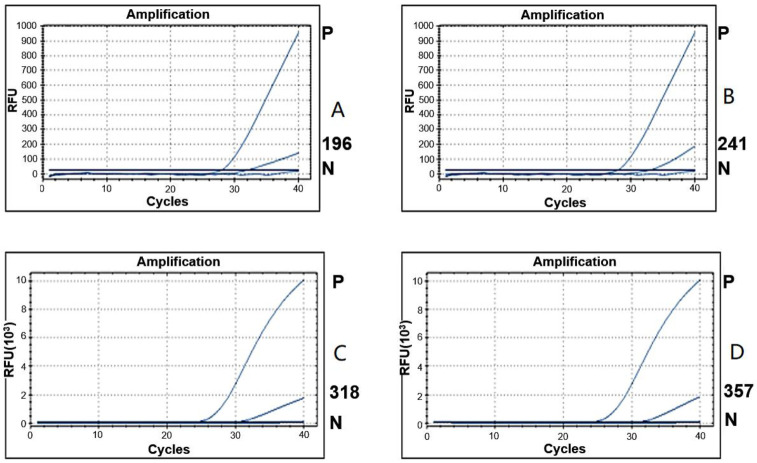
Detection of *Borrelia* spp. from ticks collected from horses and associated vegetation by real-time PCR. Amplification curves show positive detection of *Borrelia* spp. in four tick pools (196(**A**), 241(**B**), 318(**C**), and 357(**D**) ticks). Tick pool number 196 and 241 were tested together in one PCR performance, and the pool numbers 318 and 357 were done together in another PCR performance. The threshold cycle of *Borrelia* spp. detection from the four samples was 31.77; 32.81; 30.46; and 31.23, respectively. “P” and “N” represent a positive control using recombinant *B. burgdorferi* DNA and a negative control without DNA template, respectively. “RFU” indicates relative fluorescence units.

Only two, *A. phagocytophilum* and *Borrelia* spp., of the five pathogens surveyed were detected (Table 3). *Anaplasma phagocytophilum* was detected in five pools of *H. longicornis* ticks with a minimum infection rate (MIR) was 0.54%, and the pools of t *A. phagocytophilum*-positive ticks were all adults (MIR = 3.30%) (Table 3, Figure 2 and Figure 3). The number of tick pools positive for *A. phagocytophilum* collected from grasses/herbaceous vegetation associated with horse ranches and directly from horses was 3/5 (60%) and 2/5 (40%), respectively. The distributions of the infected ticks included 1/5 pools (20%) from LHR (Gyeonggi province), while the other four pools (80%) were from LHR (3), RHA (1) at Jeju Island. *Anaplasma phagocytophilum*-positive ticks from Gyeonggi province and Jeju Island were all adult *H. longicornis* ticks collected in June 2017.

*Borrelia* spp. were detected by real time PCR in four pools of ticks (MIR = 0.43%) (Table 3, Figure 3). *Borrelia* spp. was only detected in *H. longicornis* with an MIR of 0.43‰, in which three (MIR = 0.55%) and one pool (MIR = 0.66‰) were nymphs and adults, respectively. All the *Borrelia* spp. infected ticks were collected in May and June 2017, in which three pools were collected from grasses/herbaceous vegetation associated with RHA (1; Jeju Island), LHR (2; Jeollanam province (1) and Busan metropolitan city (1)), while one positive pool was collected directly from a horse at Jeju Island (Table 3).

### 2.3. Sequencing and Phylogenetic Analysis

**Figure 4:**
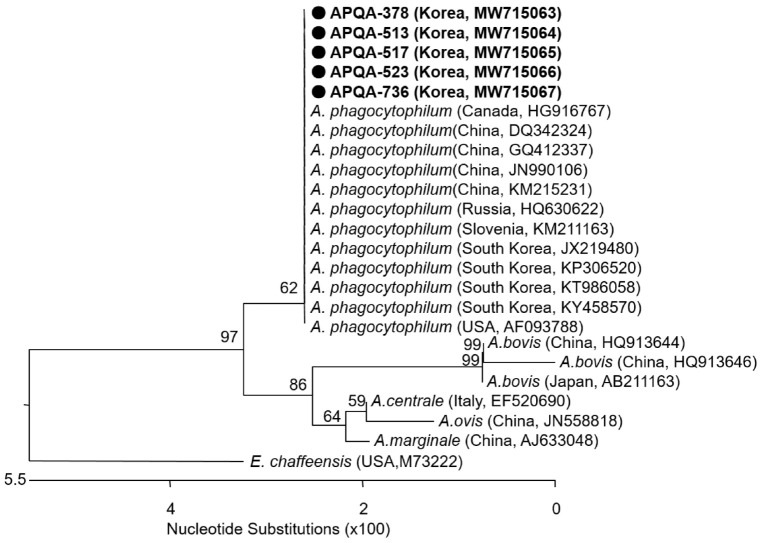
Phylogenetic relationship of *A. phagocytophilum* from ticks collected from horses and associated vegetation in the ROK. A neighbor-joining tree was created based on the 16S rRNA gene (511bp) of *A. phagocytophilum* using MEGA-6 software, with 1000 bootstrap replications. *Anaplasma phagocytophilum* detected from five tick pools collected in the ROK with isolate names of APQA-378, APQA-513, APQA-517, APQA-523, and APQA-736 and NCBI accession numbers are written in bold. The reference strains detected in different counties and the NCBI accession number are shown.

Sequencing analysis of the 16S rRNA gene of *A. phagocytophilum* from five tick samples showed 100% amino acid (aa) homology with each other and 99.78 to 100% nucleotide (nt) identity with *A. phagocytophilum* sequences deposited in the NCBI. Phylogenetic analysis based on the 16S rRNA gene (511 bp) showed that all the detected *A. phagocytophilum* had the same genotype and shared a close relationship with *A. phagocytophilum* distributed in China, USA, Canada, and Russia (Figure 4).

**Figure 5:**
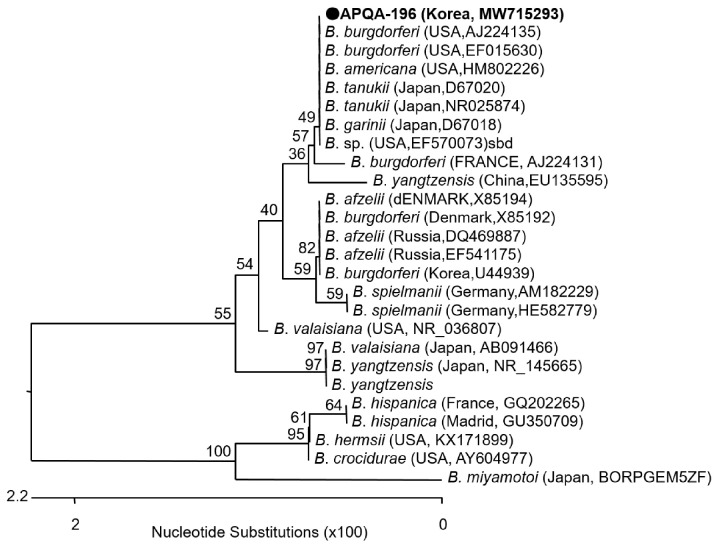
Phylogenetic relationship of *Borrelia* sp. from a pool of ticks collected from a horse in the ROK. A neighbor-joining tree was created based on the 16S rRNA gene (622 bp) of *Borrelia* sp. using MEGA-6 software, with 1,000 bootstrap replications. *Borrelia* sp. (APQA-196) detected from tick pool number 196 with NCBI accession No.: MW715293 is shown. The reference strains originated from other countries are indicated, with the NCBI accession numbers and country name are shown.

Phylogenetic analysis of *Borrelia* spp.-positive samples based on the 16S rRNA gene, showed 99.82 to 100% nt identity with reported sequences of *B. burgdorferi* listed at NCBI. The phylogenetic analysis showed a close relationship between *Borrelia* sp. in this study and strains reported from the USA (*B. burgdorferi* and *B. americana*) and Japan (*B. tanukii* and *B. garinii*) (Figure 5). The derived sequences of pathogens were submitted to the GenBank database under the accession numbers MW715063 - MW715067 (*A. phagocytophilum*) and MW715293 (*Borrelia* sp.)

## 3. Discussion

*Haemaphysalis longicornis* was the predominant tick species collected from horses in the ROK, which is consistent with previous reports that horses in the ROK are primarily infested with *H. longicornis* ticks [18,19,20]. The prevalence of ticks in the ROK was identified with a predominance of *H. longicornis*, followed by *H. flava,* and other less abundant species, such as *I. nipponensis, I. persulcatus, H. japonica*, *Amblyomma testudinarium*, and *I. granulatus* [21]. However, only two tick species, *H. longicornis* and *I. nipponensis* were detected in horses in this study. This implies that the habitat of tick species might have been affected by land use and the presence of animal reservoirs [22,23,24,25,26].

Detection of *E. chaffeensis*, *T. equi*, and *B. caballi* from cattle grazing in ROK during 2010 and 2011 showed that 19.4%, 7.2%, and 0.35%, respectively, of the tick pools were positive for the three pathogens [27], and *T. equi* infections in horses have been serologically confirmed [28]. However, the three pathogens were not detected in ticks collected from horses and associated vegetation in the ROK during 2016 and 2017 in this study. The collection of ticks and detection of associated pathogens provide information for disease risks of veterinary and medical importance and are critical for assessing disease risks and development of tick-borne disease mitigation strategies [29]. However, more rapid detection methods should be developed, such as point-of-care diagnostics from ticks collected from horses and associated vegetation, for early detection and instituting early control measure of tick-borne diseases in the future [30].

The two tick-borne pathogens, *Anaplasma phagocytophilum* and *Borrelia* spp., detected from ticks collected from horses and associated vegetation are causative agents of anaplasmosis and Lyme borreliosis in humans [31,32]. Even though the positive rate of *A. phagocytophilum*-positive ticks was very low (MIR = 0.54‰) from horse ticks in this study compared to 9.9% of ticks collected from different domestic and wild animals reported in the ROK in 2003 [33], the potential risk of transmission of this zoonotic pathogen to humans was identified [34]. Analyzing the relationship of the outbreak of human granulocytic anaplasmosis (HGA) and the collection areas of *A. phagocytophilum*-positive ticks has not been conducted due to lack of data of HGA outbreaks at areas, such as Hwasong in Gyeonggi province, and Jeju and Seoguipo cities at Jeju Island. 

*Borrelia* spp. were detected in ticks associated with horses in the ROK for the first time. While the seroprevalence of *B. burgdorferi* in horses in the ROK was 5.2% during 2009 through 2013 [35], its prevalence in ticks collected from horses and associated vegetation during 2016-2017 was very low (MIR = 0.43%). *Borrelia burgdorferi*, the causative agent of Lyme disease, is the most prevalent zoonotic TBD worldwide. Domestic animals that are susceptible to *B. burgdorferi* infections include various species, e.g., dogs, cats, horses, and ruminants [36]. In this study, *Borrelia* spp.-positive ticks were collected from vegetation associated with leisure horseback riding ranches and horse racing parks, in addition to directly from horses. However, *Borrelia* spp.-infections in horses has not been determined in this study, while human cases of Lyme disease are reported annually in Korea [26]. Therefore, there is a need to investigate the horse infectious status of *Borrelia* spp. for regions of *Borrelia* spp.-infected ticks to control and prevent zoonotic TBD in the future.

The high homology of *A. phagocytophilum* from different areas (100%) demonstrates the low variation of *A. phagocytophilum* distributed throughout the ROK. In addition, the *A. phagocytophilum* sequences detected in ticks during this study demonstrated 100% similarity to those previously detected in infected horses [37]. Various gene fragments have been used for identification of *A. phagocytophilum* [38]. However, other genes, e.g., *groEL* and *msp2*, were shown not to be helpful for the detection of *A. phagocytophilum* in the ROK [37]. 

*Borrelia* spp. were detected by specific probe-based real-time PCR and then confirmed based on sequencing analysis of the 16S rRNA gene. However, sequence results of 16S rRNA gene was not useful for phylogenetic identification of *B. burgdorferi* sensu lato because the sequence also shared 100% identity to *B. tanukii*. Therefore, further analysis using various primer sets of alternate gene fragments [39,40], and specific primers for each species detection are necessary. Unfortunately, the nucleic acids extracted from the positive samples were exhausted. Therefore, we could not conduct further analysis for the detected *Borrelia* spp. in this study.

A nationwide surveillance of tick prevalence and tick-borne pathogens harbored by horse ticks was conducted in this study for the first time. The result revealed that horses in the ROK are infested by two tick species, *H. longicornis* and *I. nipponensis,* with majority of *H. longicornis* (99%). These ticks are vectors of two important tick-borne pathogens, *A. phagocytophilum* and *Borrelia* spp., among the selected five targets for detection (*A. phagocytophilum*, *E. chaffeensis*, *Borrelia* spp., *B. caballi*, and *T. equi*). The survey of tick-borne pathogens harbored by horse ticks should be further extended for other important pathogens, such as *Rickettsia*, by which a strategy for diagnosis and prevention of the related diseases could be established.

## 4. Materials and Methods

### 4.1. Tick Collection and Identification

**Figure 6:**
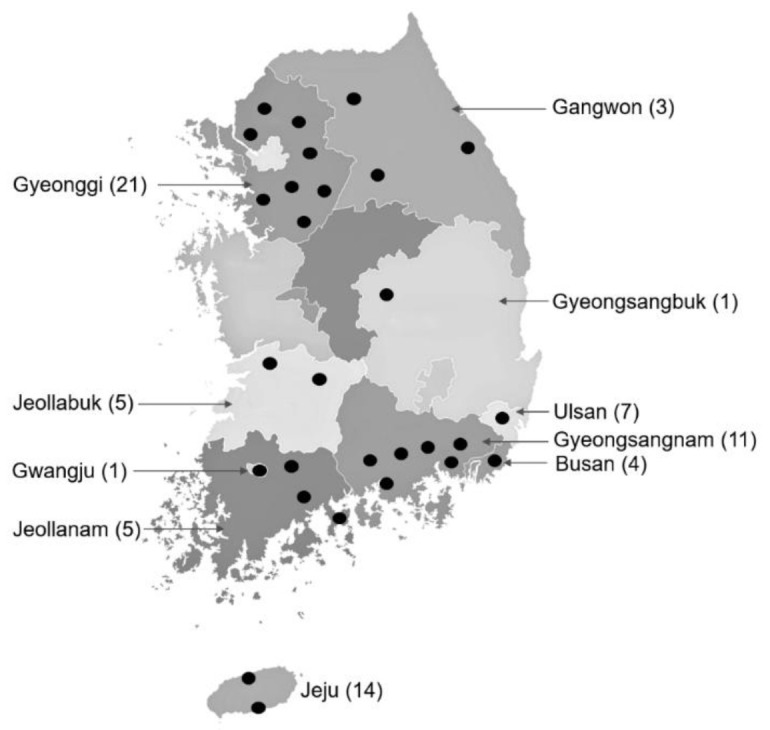
Collection sites of ticks from horses and associated vegetation at horse ranches and racetracks in the ROK. Collection sites in each province or metropolitan city are indicated by black circles. Number of ranches where samples were collected in each province or metropolitan city are shown in parentheses.

Ticks were collected directly from horses and associated vegetation at 72 sites, including horse racetracks (2) and stud farms (3) operated by the Racing Horse Authority, PHF (11), and LHR (56) in the ROK. Twenty four of the ranches were located in Gyeonggi and Gangwon provinces in Northern ROK, while the other 48 ranches were located in central (2) and southern (46) provinces and metropolitan cities (Figure 6). Ticks on horses were removed by securing the mouthparts with fine forceps as close to the skin as possible and gently pulling the tick away to avoid breaking off the mouthparts, while ticks were collected from the vegetation by the dragging/flagging method. Ticks were placed in 15 mL or 50 mL plastic vials with screw tops. At the end of each collection, the ticks were placed in a cooler where they were transported to the Parasitic and Honeybee Disease Laboratory, the Animal and Plant Quarantine Agency and stored at −80 °C until further identified.

Sex determination, identification of species and developmental stages were carried out using morphological keys [41,42,43] under a light for all tick individuals, and a subset of ticks was identified using electron microscopy. After identification, the ticks were transferred to 1.5 mL cryovials according to species, stage of development, and sex (adults) and returned to the −80 °C freezer until they were processed for the detection of selected tick-borne agents.

### 4.2. Extraction of Nucleic Acids

Ticks were pooled based on the collection date, location, species, developmental stage, and sex. Each pool consisted of adults (1–5), nymphs (1–30), or larvae (1–50). Ticks from each pool and 300 µL of PBS solution were added in a tissue-homogenizing tube with steel beads (SNC, Hanam, Korea), the sample was homogenized using a Precellys 24 Tissue Homogenizer (Bertin Instruments, Montigny-le-Bretonneux, France). Maxwell RSC Viral Total Nucleic Acid Purification Kit (Promega, Madison, WI, USA) was used for total nucleic acids extraction. The homogenate, 300 µL of lysis buffer, and 30 µL of proteinase K solution were added in a new 1.5 mL microcentrifuge tube. After incubating at 56 ℃ for 10 min purification of nucleic acids was done by using an automated Maxwell RSC Instrument (Promega, Madison, WI, USA). Isolated material was stored at −80 °C until further molecular analysis.

### 4.3. Polymerase Chain Reaction (PCR) and Real-Time PCR

The detection of *Babesia* spp., *B. caballi*, *T. equi*, *A. phagocytophilum*, and *E. chaffeensis* was performed using conventional PCR, and the AccuPower ProFi Taq PCR PreMix (Bioneer, Daejeon, Korea). Each 20 µL reaction mix included 5 µL DNA template, 1 µL (10 pmol) of each primer, 13 µL of double-distilled water (ddH_2_O). The PCR conditions used to amplify each target are shown in Table 4. Detection of *Borrelia* spp. was performed using real-time PCR (CFX96 Touch Real-time PCR Detection System; Bio-Rad Laboratories, Inc., Hercules, CA). Each 20 µL reaction mixture consisted of 1 µL (10 pmol) of each primer, 1 µL (5 pmol) of probe, 10 µL of PCR premix (IQ supermix, Bio-Rad Laboratories, Inc., Hercules, CA, USA), 5 µL of DNA template, and 2 µL of ddH_2_O (Table 4). The volume of DNA template (5 µL) used for each PCR was examined without PCR inhibition (Supplementary Figure S1).

Results of positive detections were expressed as a minimum infection rate (MIR) that assumed that every positive pool contains only one infected tick. The MIR was calculated using the formula: MIR = number of positive pools/total number of tested ticks × 1000 [44,45].

### 4.4. Phylogenetic Analysis

Positive samples of *Borrelia* spp. detected by real-time PCR were analyzed phylogenetically using the 16S rRNA gene and PCR products (622 bp; Table 4) amplified and sequenced. Phylogenetic analysis of *A. phagocytophilum* was performed using the 16S rRNA gene and PCR products of positive samples purified using a QIA Quick Purification Kit (Qiagen, Hilden, Germany) and Macrogen (Seoul, Korea) sequenced the PCR products. The homologies of the generated sequences were analysed using the BLASTn tool of the National Center for Biotechnology Information (NCBI) GenBank database. The sequences were aligned using the Clustal W with MegAlign software version 7.1 (DNA-STAR, Madison, WI, USA) and phylogenetic trees generated using the neighbor-joining algorithm in MEGA-6 software [46] with 1000 bootstrap replications.

## Figures and Tables

**Table 1:** Number of ticks collected (pools) at selected metropolitan areas and provinces.

Metropolitan City and Province	Collecting Source	2016				2017			Total
		H. longicornis *			I. nipponensis *	H. longicornis *			
		Larva	Nymph	Adult	Adult	Larva	Nymph	Adult	
Busan	Horse	0	0	0	0	0	0	0	0
	Vegetation	2(1)	2(1)	0	0	0	148(17)	8(8)	160(27)
	Subtotal	2(1)	2(1)	0	0	0	148(17)	8(8)	160(27)
Gwangju	Horse	0	0	0	0	0	0	0	0
	Vegetation	1(1)	1(1)	0	0	0	65(7)	5(5)	72(14)
	Subtotal	1(1)	1(1)	0	0	0	65(7)	5(5)	72(14)
Ulsan	Horse	0	0	4(1)	0	0	0	0	4(1)
	Vegetation	81(2)	101(7)	14(4)	0	0	0	0	196(13)
	Subtotal	81(2)	101(7)	18(5)	0	0	0	0	200(14)
Gangwon	Horse	0	0	0	0	0	0	0	0
	Vegetation	293(7)	5(2)	0	0	0	0	0	298(9)
	Subtotal	293(7)	5(2)	0	0	0	0	0	298(9)
Gyeonggi	Horse	0	9(3)	0	0	0	0	0	9(3)
	Vegetation	0	134(9)	0	4(3)	81(3)	375(45)	11(11)	605(71)
	Subtotal	0	143(12)	0	4(3)	81(3)	375(45)	11(11)	614(74)
Gyeongsangbuk	Horse	0	0	33(11)	0	0	0	0	33(11)
	Vegetation	60(2)	128(5)	32(7)	0	0	0	0	220(14)
	Subtotal	60(2)	128(5)	65(18)	0	0	0	0	253(25)
Gyeongsangnam	Horse	0	0	3(2)	0	0	0	0	3(2)
	Vegetation	0	0	0	0	0	269(27)	8(8)	277(35)
	Subtotal	0	0	3(2)	0	0	269(27)	8(8)	280(37)
Jeollabuk	Horse	1(1)	126(42)	12(5)	2(1)	0	0	0	141(49)
	Vegetation	0	108(8)	2(2)	0	0	3(2)	2(2)	115(14)
	Subtotal	1(1)	234(50)	14(7)	2(1)	0	3(2)	2(2)	256(63)
Jeollanam	Horse	0	0	0	0	0	0	0	0
	Vegetation	2(1)	6(2)	0	0	0	361(38)	11(11)	380(52)
	Subtotal	2(1)	6(2)	0	0	0	361(38)	11(11)	380(52)
Jeju Island	Horse	0	454(156)	645(219)	0	0	6(3)	390(282)	1495(660)
	Vegetation	1746(37)	2108(78)	156(43)	0		960(99)	168(168)	5138(425)
	Subtotal	1746(37)	2562(234)	801(262)	0	0	966(102)	558(450)	6633(1085)
Total	Horse	1(1)	589(201)	698(237)	2(1)	0	6(3)	390(282)	1686(725)
	Vegetation	2185(51)	2657(121)	213(62)	4(3)	81(3)	2181(235)	213(213)	7534(688)
	Subtotal	2186(52)	3246(322)	911(299)	6(4)	81(3)	2187(238)	603(495)	9220(1413)

Note: “*” number of ticks with pool number was shown in parentheses.

**Table 2:** Identification of tick species from horses and vegetative at racetracks and private horse ranches.

Spots	Colletion Source	2016				2017			Total
		H. longicornis **			I. nipponensis **	H. longicornis **			
		Larva	Nymph	Adult	Adult	Larva	Nymph	Adult	
RHA *	Horse	1(1)	139(47)	74(30)	2(1)	0	0	211(111)	427(190)
	Vegetation	2(1)	324(18)	11(6)	2(1)	0	678(68)	88(88)	1105(182)
	Subtotal	3(2)	463(65)	85(36)	4(2)	0	678(68)	299(199)	1532(372)
PHF	Horse	0	263(90)	220(78)	0	0	0	0	483(168)
	Vegetation	63(3)	1435(54)	97(28)	1(1)	0	101(12)	4(4)	1701(102)
	Subtotal	63(3)	1698(144)	317(106)	1(1)	0	101(12)	4(4)	2184(270)
LHR	Horse	0	187(64)	404(129)	0	0	6(3)	179(171)	776(367)
	Vegetation	2120(47)	898(49)	105(26)	1(1)	81(3)	1402(155)	121(121)	4728(402)
	Subtotal	2120(47)	1085(113)	509(155)	1(1)	81(3)	1408(158)	300(292)	5504(769)
Total	Horse	1(1)	589(201)	698(237)	2(1)	0	6(3)	390(282)	1686(725)
	Vegetation	2185(51)	2657(121)	213(62)	4(3)	81(3)	2181(235)	213(213)	7534(688)
	Subtotal	2186(52)	3246(322)	911(299)	6(4)	81(3)	2187(238)	603(495)	9220(1413)

* RHA, Racetracks under the Racing Horse Authority; PHF, private horse farms; LHR, leisure horse-riding ranches. “**” number of ticks with pool number shown in parentheses.

**Table 3:** Tick-borne pathogens detected in ticks collected from horses in the Republic of Korea.

Year	Species	Stage	Tick No. (Pool)	No. of PCR Positive Tick Pools				
				*B. caballi*	*T. equi*	*A. phagocytophilum*	*E. chaffeensis*	*B. burgdorferi s.l.*
2016	*H. longicornis*	Larva	2186 (52)	0	0	0	0	0
		Nymph	3246 (322)	0	0	0	0	0
		Adult	911 (299)	0	0	0	0	0
		Subtotal	6343 (673)	0	0	0	0	0
	*I. nipponensis*	Adult	6 (4)	0	0	0	0	0
2017	*H. longicornis*	Larva	81 (3)	0	0	0	0	0
		Nymph	2187 (238)	0	0	0	0	3
		Adult	603 (495)	0	0	5	0	1
		Subtotal	2871 (736)	0	0	5	0	4
Total			9220 (1413)	0	0	5	0	4

**Table 4:** PCR primer sets and conditions used for detecting tick-borne pathogens in ticks collected from horses and associated vegetation.

Pathogens	Primers	Sequences (5′-3′)	PCR Condition	Target Gene (bp)	Reference
*B. caballi/* *T. equi*	Bec-UF1	GTTGATCCTGCCAGTAGTCA	95 °C (5 min); 37 cycles of 95 °C (30 s), 56 °C (30 s), and 72 °C (1 min)	*18S rRNA* (*Bc*:867/ *Te*:913)	[47]
	Bec-UR	CGGTATCTGATCGTCTTCGA			
*Babesia* spp.	B5UK1	GTCAGCAAGCGAACCGACAA	95 °C (5 min); 37 cycles of 95 °C (30 s), 55 °C (30 s), and 72 °C (40 s)	*SpS7* (670)	[48]
	B3UK1	CCAAGACGAGCTGAAGGATC			
*Anaplasma phagocytophilum*	PITA-fwd	GTCGAACGGATTATTCTTTA	95 °C (5 min); 37 cycles of 95 °C (30 s), 58 °C (30 s), and 72 °C (40 s)	*16S rRNA* (511)	[49]
	PITA-rev	TTCACCTTTAACTTACCGAA			
*Ehrlichia chaffeensis*	HE1	CAATTGCTTATAACCTTTTGGTTATAAAT	95 °C (5 min); 37 cycles of 95 °C (30 s), 50 °C (30 s), and 72 °C (30 s)	*16S rRNA* (390)	[50]
	HE3	TATAGGTACCGTCATTATCTTCCCTAT			
*Borrelia* spp.	Bb23Sp-FAM	56-FAM/AGATGTGGT/ZEN/AGACCCGAAGCCGAGTG/31ABkFQ	50 °C (2 min); 95 °C (5 min); 40 cycles of 95 °C (15 s) and 60 °C (30 s)	*23S rRNA* (75)	[51]
	Bb23Sf	CGAGTCTTAAAAGGGCGATTTAGT			
	Bb23Sr	GCTTCAGCCTGGCCATAAATA			
	Bb16s-F	GAGGCGAAGGCGAACTTCTG	95 °C (5min); 37 cycles of 95 °C (30 s), 60 °C (30 s), and 72 °C (1 min)	*16S rRNA* (622)	[52]
	Bb16s-R	CTAGCGATTCCAACTTCATGAAG			

## Data Availability

The data presented in this study are available on request from the corresponding author.

## Supplementary Materials

The following are available online at https://www.mdpi.com/article/10.3390/pathogens10091069/s1, Figure S1: Checking PCR inhibition of tick nucleic acids solution.
