# Supplementary material for: Molecular Detection and Phylogenetic Analysis of Tick-Borne Pathogens in Ticks Collected from Horses in the Republic of Korea
Source: Pathogens. 2021 Aug 24;10(9):1069. doi: 10.3390/pathogens10091069 (PMC8472514; doi:10.3390/pathogens10091069)
Supplement: Supplementary file 1 [file pathogens-10-01069-s001.zip › pathogens-1288510-supplementary.pdf]

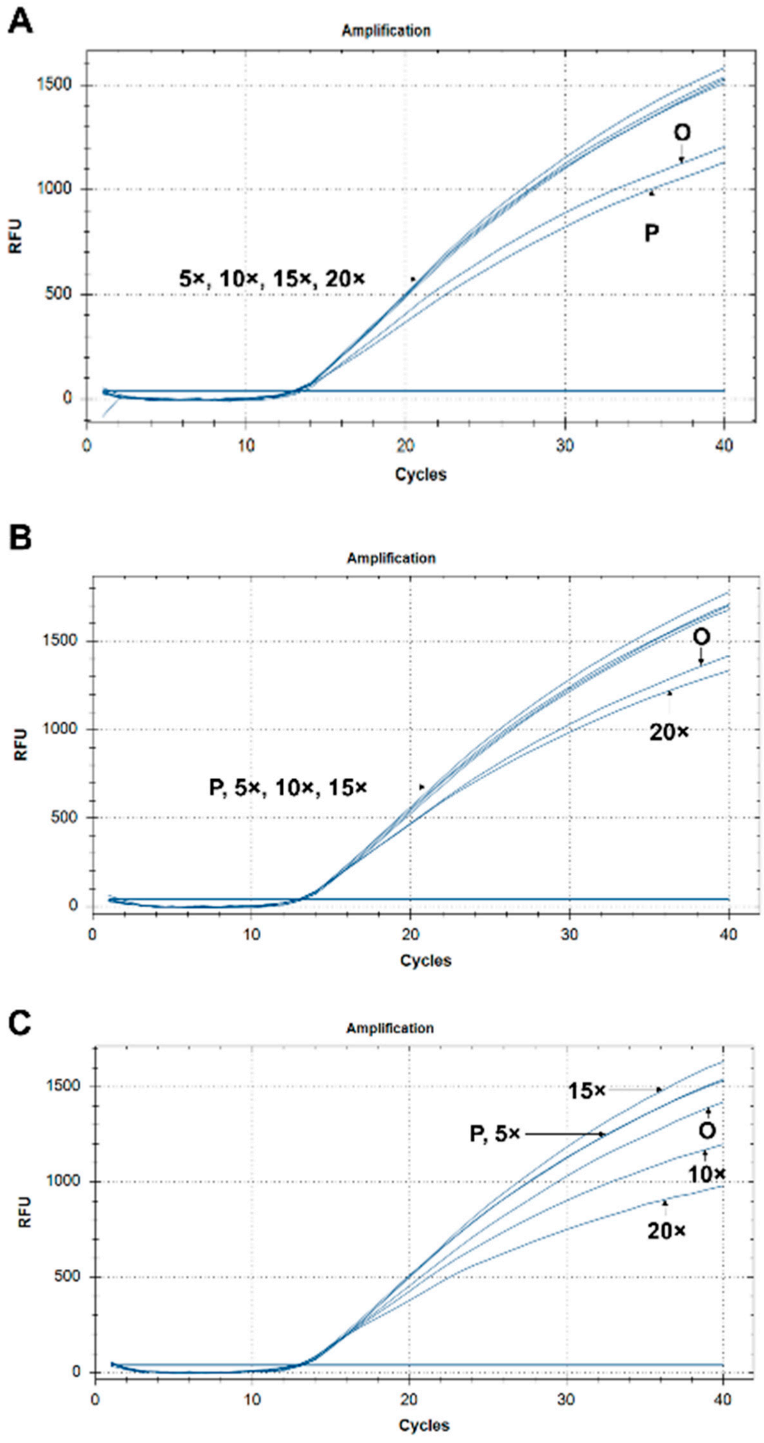

Figure S1. Checking PCR inhibition of tick nucleic acids solution. *Coxiella burnetii* recombinant DNA (2.8×10^8 copies) and 5µL of tick nucleic acids extracted from one adult tick (A), 10 nymphs (B), and 50 larvae (C) were used for each reaction to examined the PCR inhibition of tick nucleic acids. "P" indicates the reaction using only *Coxiella burnetii* recombinant DNA (2.8×10^8 copies) without tick nucleic acids, "O" indicates the reaction using *Coxiella burnetii* recombinant DNA (2.8×10^8 copies) and 5 µL of tick nucleic acids without dilution, "5 ×, 10 ×, 15 ×, and 20 ×" indicate reaction using 2.8×10^8 copies of *C. burnetii* recombinant DNA and 5 µL of tick nucleic acids with 5, 10, 15, and 20 times dilution, respectively. The threshold cycle of amplification was not great different among the reaction. Therefore, the tick nucleic acids with 5µL used in each reaction could not greatly inhibit the detection of target pathogens in this study.
